# Cross-species transcriptional network analysis reveals conservation and variation in response to metal stress in cyanobacteria

**DOI:** 10.1186/1471-2164-14-112

**Published:** 2013-02-19

**Authors:** Jiangxin Wang, Gang Wu, Lei Chen, Weiwen Zhang

**Affiliations:** 1 School of Chemical Engineering & Technology, Tianjin University, 300072, Tianjin, People's Republic of China; 2 Key Laboratory of Systems Bioengineering, Ministry of Education, 300072, Tianjin, People's Republic of China; 3 Department of Biological Sciences, University of Maryland at Baltimore County, 21250, Baltimore, MD, USA

**Keywords:** Cross-species, Transcriptional network, Metal stress, Cyanobacteria

## Abstract

**Background:**

As one of the most dominant bacterial groups on Earth, cyanobacteria play a pivotal role in the global carbon cycling and the Earth atmosphere composition. Understanding their molecular responses to environmental perturbations has important scientific and environmental values. Since important biological processes or networks are often evolutionarily conserved, the cross-species transcriptional network analysis offers a useful strategy to decipher conserved and species-specific transcriptional mechanisms that cells utilize to deal with various biotic and abiotic disturbances, and it will eventually lead to a better understanding of associated adaptation and regulatory networks.

**Results:**

In this study, the Weighted Gene Co-expression Network Analysis (WGCNA) approach was used to establish transcriptional networks for four important cyanobacteria species under metal stress, including iron depletion and high copper conditions. Cross-species network comparison led to discovery of several core response modules and genes possibly essential to metal stress, as well as species-specific hub genes for metal stresses in different cyanobacteria species, shedding light on survival strategies of cyanobacteria responding to different environmental perturbations.

**Conclusions:**

The WGCNA analysis demonstrated that the application of cross-species transcriptional network analysis will lead to novel insights to molecular response to environmental changes which will otherwise not be achieved by analyzing data from a single species.

## Background

Many biological systems operate in a similar manner across a large number of species or conditions [1]. Cross-species analysis of genomic sequences has made fundamental contributions to modern biology in defining the putative function of new genes [2]. Recent exponential growth in microarray expression datasets allows researchers to combine expression experiments from multiple species to identify genes that are not only conserved in sequence but also operated in a similar way [3]. In contrast to the static sequence measurements, microarrays measure the dynamic, condition-specific responses of complex biological systems [4-7], and their comparative analysis has led to improvements in annotating gene function and inferring the evolution of interaction and regulatory networks [3]. In addition, cross-species microarray analysis also allowed identification of core transcriptional networks, and their conservation and variation in closely related species [8,9].

Correlation networks are increasingly being used in various bioinformatics applications [10-12]. Among them, Weighted Gene Co-expression Network Analysis (WGCNA) is a computational method to describe the correlation patterns among genes across transcriptomic datasets [11,13]. WGCNA has been used in identifying functional clusters (modules) of highly correlated genes, summarizing such clusters using the module eigengene or an intramodular hub gene, relating modules to one another and to external sample traits (using eigengene network methodology), calculating module membership measures in many systems, and correlation network analysis can also be used to determine biological correlated candidate biomarkers or disease therapeutic targets [11]. The WGCNA approach has been applied in many eukaryotic systems, successfully linking molecular targets to oncogenic signals [14], complex traits [15], analyzing network divergence between human and chimpanzee neural patterns [16], and even comparing cross-species gene expression in animals recently [17]. A recent comparative study of different network analysis methods indicated that WGCNA could be used not only for constructing gene networks, but also for detecting modules/sub-networks, identifying hub genes and selecting candidate genes as biomarkers, using *Escherichia coli* as an empirical sample [18].

As important primary producers and significant contributors of fixed carbon budget in many terrestrial and marine environments, cyanobacteria have been present in many different environments from coast to open sea for ~2.5 billion years [19]. Currently the marine cyanobacteria, *Synechococcus* and *Prochlorococcus* species are together responsible for at least 20% of global carbon fixation [20]. They play such significant roles in the carbon cycle that it is essential to understand to what environmental stresses they are susceptible and how they respond. Metal is one important environmental factor for cyanobacteria. For instance, iron is required for photosynthesis, and its limitation and restriction of primary productivity have been reported and has been considered as one important factor in the ecology of cyanobacteria [21,22]. Early studies have found the limitation of *Prochlorococcus* cell division rates by iron in the equatorial Pacific sea [23], and variations in the abundance of *Prochlorococcus* iron-related genes between oceans [24]. A recent study on transcriptomic response of high- and low-light-adapted *Prochlorococcus* strains to changing iron availability suggested high divergence of gene expression within strains [25]. The physiology and biochemistry associated with the iron-limited continuous culture of the halotolerant cyanobacterium *Synechococcus* PCC 7002 were also examined [26], and the regulatory network for acclimation of the obligate photoautotrophic fresh water cyanobacterium *S. elongatus* PCC 7942 to iron limitation was studied by transcript profiling recently [27]. Interestingly, oceanic *Synechococcus* strains were found much more sensitive to iron limitation than coastal strains [28]. Copper is another metal commonly present in natural environments. It has been reported that different cyanobacteria species have varying levels of copper tolerance, with *Prochlorococcus* species considered to be more sensitive to copper than *Synechococcus* species [29]. Within *Synechococcus* species, copper tolerance has also varied significantly [30], with coastal species of *Synechococcus* species exhibiting increased tolerance to copper shock and a distinctive transcriptional responses relative to those of open-ocean species [31].

To seek better understanding of molecular response of cyanobacteria to metal stress, as a proof of concept we applied a cross-species transcriptional network analysis to four important cyanobacteria species under two different conditions of metal stress, *Prochlorococcus* MED4 (PMM) and *Prochlorococcus* MIT9313 (PMT) under stress of iron depletion [25], *Synechococcus* 9311 (SYG) and *Synechococcus* WH 8102 (SYW) under stress of high copper concentration [31]. Transcriptional networks for each individual species were constructed using a WGCNA method [11,13], and then cross-strain analysis was performed to reveal the conservation and variation in terms of the response to iron and copper stresses among all species. In this study, cross-species analyses of two *Synechococcus* species under high copper stress condition, and two *Prochlorococcus* species under iron depletion stress condition were first performed separately, the results revealed the signature responses to iron in *Synechococcus* and to copper in *Prochlorococcus*, respectively. Then a cross-species comparison was conducted for all four cyanobacteria species under two different types of metal stresses. Notably, the results showed that 9 genes were commonly regulated in all four species used in this study. Although still needs more experimental evidences, the set of genes may represent an important core signature response to metals in these four cyanobacterial species. Interestingly, the species-specific hub genes detected in four species showed no overlap between each other, indicating possible species-specific strategy for metal acclimation in each species. Thus, the analysis demonstrated that the application of cross-species transcriptional network analysis could lead to novel insights into molecular response to environmental changes which will otherwise not be achieved by analyzing data from a single species.

## Results and discussion

### Construction of cyanobacteria metal response networks

Comparison of transcriptional networks between different cyanobacteria species could provide valuable insights into the core and important responses to environmental stress at the molecular levels. To this end, we first sought to compile a complete transcriptional profiling datasets of different cyanobacteria species under iron depletion and copper toxicity. The datasets used in our analysis included transcriptional measurements of a total of 50 samples from 4 cyanobacteria species (*i.e.* PMM, PMT, SYG, SYW), representing iron depletion (PMT and PMM) and high copper toxicity (SYG and SYW) treatments, respectively (Table 1) [25,31]. To ensure that our dataset were reasonably selected, we also compared the treatment sample datasets with their controls through a clustering analysis. The results showed that all stress-treated datasets tended to be grouped together and can be visibly separated from the controls, suggesting the stresses of iron depletion and copper toxicity have caused significant changes at the transcriptional level (Additional file 1: Figure S1). Next, we applied the datasets of each species for the transcriptional network construction using a WGCNA method. Analysis of the transcriptional networks showed that a total of 17, 21, 32 and 28 distinct transcriptional modules can be detected within the transcriptional networks of PMT, PMM, SYG and SYW, respectively (Additional file 2: Figure S2). More transcriptional modules detected in SYG and SYW than PMT and PMM were probably due to the relatively large genome sizes of *Synechococcus*[32-34]. The association analysis between the phenotypes (*i.e.* iron or copper stress) and the detected modules showed that the distinguished transcriptional modules highly associated with the phenotypes can be identified in each of the four species according to the correlation coefficients (*r* value) and their confidence (low *p*-values) (Additional file 3: Figure S3). Among all transcriptional modules detected in each species, we found that 5 of 17 and 6 of 21 detected modules significantly correlated with iron depletion condition in PMT and PMM, respectively (Table 2); and 12 of 32 and 11 of 28 detected modules significantly correlated with the high copper toxicity in SYG and SYW, respectively (Table 2). The KEGG pathway analysis of these phenotype-correlated modules in different species showed that a total of 62-79 KEGG pathways were involved in these modules (Table 2). Interestingly, 69 and 49 KEGG shared pathways were detected in PMT/PMM to iron depletion and SYG/SYW to high copper toxicity, respectively (Table 2).

**Table 1:** Cyanobacteria species and related datasets used in this study

**Species**	**Code**	**Condition (GEO ID)**	**Original sample size**	**Included in this study after filtering**
***Synechococcus*9311**	SYG	Copper toxicity (GSE13910)	14	14
***Synechococcus*WH 8102**	SYW	Copper toxicity (GSE13910)	10	10
***Prochlorococcus*MED4**	PMM	Iron depletion (GSE26533)	16	10
***Prochlorococcus*MIT 9313**	PMT	Iron depletion (GSE26533)	23	16

**Table 2:** Modules and KEGG pathways significantly responsive to the metal treatments

**Metal stress**	**Species**	**Modules related to phenotype**	**KEGG Pathways**	**Common pathways**
**Iron**	PMT	5	79	65
	PMM	6	68	
**Copper**	SYW	12	63	49
		SYG	11	62

Several cyanobacteria species produce secondary metabolites such as xanthophyll carotenoids and their synthetic genes are often up-regulated in response to high light intensity, ultraviolet radiation, and desiccation [35]. The ability to exist in two redox states makes iron an essential cofactor for proteins involved in numerous major cellular processes such as respiration, amino acid metabolism and DNA metabolism. For instance, iron starvation down-regulated most amino acid biosynthesis related genes since many enzymes are iron dependent [36,37]. It was also reported that iron starvation also induced DNA recombination and DNA repair process in a Gram-negative diplococci bacteria [38]. PMT was originally isolated from the gulfstream 135 meter in depth (low light adapted) and could grow with iron of an order of magnitude lower [25]. Analysis of the iron-responsive modules suggested that the significant down-regulation of “amino acid metabolism” and “microbial metabolism in diverse environments” probably reduced the iron requirement, and the up-regulation of the “metabolism of secondary metabolites” protected the cells from oxidative stress caused by iron depletion directly or indirectly. At the same time, the up-regulation of “homologous recombination” might result in an increase of DNA mismatch repair and recombination [39]. Detailed analysis of genes with the same change trends (up- or down-regulated) in both PMM and PMT under iron depletion condition suggested the mechanism that cyanobacteria employ to iron deficiency could be: firstly transporters were activated to obtain more iron from the environment [25], and photosynthesis and chlorophyll metabolism was inhibited due to the limited supply of iron [40-42], and then oxidative stress was enhanced and may cause RNA degradation, protein translation inhibition and other cellular metabolic changes. All these further resulted in inhibition of DNA replication and cell cycle block, and eventually growth cease.

The copper-responsive SYW yellow module showed a significantly high correlation between “ribosome biosynthesis and assembly” (syw03010, total 20 related genes enriched) and the copper toxicity treatments (*p* = 6.78E-15), suggesting an involvement of protein synthesis program in copper acclimation (Additional file 4: Table S1). Another copper-responsive SYW greenyellow module showed a significant correlation with the “aminoacyl-tRNA biosynthesis” and “citrate cycle” (TCA cycle) pathways, which was characterized as a down-regulation response. According to KEGG pathway analysis, genes in this module tended to encode proteins involved in amino acid metabolism (*p* = 0.0012~0.0372) (Additional file 5: Table S2). The genes involved in “polycyclic aromatic hydrocarbon degradation” were identified in the copper-responsive SYG darkgreen module. The module also showed a significant correlation with amino acid metabolism (histidine, tyrosine, cysteine and methionine, arginine and proline; *p* = 5.59E-05~0.005). KEGG pathway analysis indicated that the SYG darkgreen module was enriched with genes encoding photosynthesis antenna proteins, and genes related to “microbial metabolism in diverse environments” and “oxidative phosphorylation” (Additional file 6: Table S3). The copper-responsive SYG brown module showed significant correlation with the “curated two-component”, overrepresented with genes participating in signal transduction activity, including “response regulator”, “two component system”, “histidine kinase” and “ABC transporters”. (Additional file 7: Table S4), suggesting the strong involvement of signal transduction systems in copper response in SYG species.

For high copper toxicity, a recent study showed that costal strains of marine *Synechococcus* species (SYG in this study) exhibited increased tolerance to copper shock and a distinctive transcriptional response relative to those of open-ocean strains (SYW) [31]. SYG was predicated to be more metal-tolerant and may be better in adapting to fast changing environments, including biotic and abiotic environmental stresses besides copper. Early studies showed that toxicity of excess copper may be arose through several mechanisms: production of reactive oxygen species (ROS), metal competitive binding and photosynthesis inhibition [31,43,44]. In this study, we found that significant copper-responsive modules existed in both species of SYG and SYW. Modules of SYW greenyellow and SYG darkgreen both contained genes related to “amino acid metabolism” and “microbial metabolism in diverse environments”, representing a general stress response. Meanwhile, more specific responses were also found in SYW yellow module which contained a down-regulated cluster of ribosomal genes, and in SYG brown module which had up-regulated signal transduction and response, such as “curated two-component system”, RR and HK system. The overlap of orthologous genes between SYW and SYG was modest, with only 46 orthologous genes being significantly regulated by high copper. Based on these results, we proposed that a possible strategy for SYW cells to survive under high copper condition was to slow down *de novo* protein synthesis and employ oxidative protection to eliminate the ROS damage caused by copper treatment, while SYW cells may use relatively active signal transduction and response systems to adjust its metabolism to adapt the changing environments.

### Signature genes and pathways of different cyanobacteria species under metal stresses

Using WGCNA, we obtained modules that contained an exact number of assigned genes. The same genes could be assigned to multiple modules, although the association strengths in different modules could vary. Our results showed a high degree of cross-species module similarity between PMM and PMT under iron depletion condition, and between SYW and SYG under high copper condition, suggesting possible core responses to each treatment. In addition, the overlaps between iron depletion and copper toxicity response modules were also observed, suggesting the possible core and general response to metals and possible cross-talking response networks between these four species to different metal stresses. We identified the shared genes between response modules in different cyanobacteria species based on the significance of module membership values (Additional file 8: Figure S4, Additional file 9: Table S5 and Additional file 10: Table S6). The results showed that a total of 440 and 430 genes were found correlated with iron depletion in PMM and PMT, respectively, with only 34 of them shared between PMM and PMT with confident low *p*-values (Additional file 8: Figure S4A). Similarly, the numbers of the copper correlated genes were 736 and 580 in SYG and SYW, respectively (Additional file 8: Figure S4B), with only 70 of them shared between SYG and SYW.

In a recent study, only 4 of 1159 orthologous genes of PMT and PMM were found differentially regulated in response to iron in both species (using fold change > 2.0), although expression level of over a hundred genes changed in two species, suggesting a great diversity in terms of iron adaptation among different *Prochlorococcus* species [25]. Interestingly, only 1 pair (*petF*, ferredoxin, PMM0898/PMT1429) of 4 differentially expressed genes (*petF*, *isiB* PMM1171/PMT0801, *hli05/08* PMM1404/PMT1154, and *idiA* PMM1164/PMT0287) in iron stress previously reported [25] was showed in our shared gene list, due to the low correlation coefficient of the other three genes in the modules with *r* ranging from 0.001 to 0.42. Conventionally for microarray analysis, a cutoff of >2-fold change was commonly used; it is quite possible that some of the genes with important functions will have a smaller change (fold change < 2.0) and thus will be excluded. In this study, we focused on the co-expression property across two different species, thus no fold change threshold was applied, by doing this we could avoid information loss due to artificial fold-change cutoff (*i.e.* genes changed with significantly low *p* values, but with less than 2.0 fold changes). Instead, we focused on only those genes in highly correlated modules to explore their possible functions in metal acclimation.

Interestingly, our cross-species analysis revealed 9 commonly shared genes among these four cyanobacteria species under two different metal stress conditions (Table 3). They were ABC transporter ATP binding component, carboxypeptidase Taq metallopeptidase, cell division protein (*ftsW*), ferredixin, isocitrate dehydrogenase (NAD^+^) (*idh3*), LysM domain, phosphoribosylaminoimidazolecarboxamide formyltransferase/IMP cyclohydrolase (*purH*), phycocyanobilin:ferredoxin oxidoreductase (*pcyA*) and 6-pyruvoyl tetrahydrobiopterin synthase (*pts*) (Table 3). However, not all of these genes shared the same expression patterns cross species, except for one gene, *ftsW*, which was down-regulated in all four species under both iron starvation and copper toxicity. Encoding a cell-division intergral membrane protein, *ftsW* has been found essential in *E. coli*[45] and was required for Z-ring stabilization during sporulation septation in *Streptomyces coelicolor*[46]. Both FtsQ (another cell division protein) and FtsW are indispensable to *Synechocystis* and their depletion led to slow growth and giant cells [47]. Recent study indicated that FtsW was also directly involved in DNA damage checkpoint coordinately interacting with an SOS regulon in *Caulobacter crescentus*[48], and the down-regulation of this gene might inhibit cell division [49]. Both iron depletion and high copper can cause oxidative stress and DNA damage [25,31], it could thus be speculated that *ftzW* gene may play an important role in cellular defense to environmental disturbances.

**Table 3:** Genes responsive in all four cyanobacteria species

**KO_ID**	**Name**	**Taxon**	**Gene**	**Module**	**Module cor**	**Fold change (log2)**	**p-value**
K01737	6-pyruvoyl tetrahydrobiopterin synthase	PMM	PMM0106	turquoise	-0.755	-0.5	0.024
		PMT	PMT0164	turquoise	-0.611	0.3	0.0009
		SYW	SYNW1504	black	0.791	1.5	0.013
		SYG	sync_1898	brown	0.977	0.2	0.002
K00602	phosphoribosylaminoimidazolecarboxamide formyltransferase / IMP cyclohydrolase	PMM	PMM0266	magenta	0.714	0.3	0.028
		PMT	PMT1857	purple	0.613	0.3	0.025
		SYG	sync_0289	turquoise	-0.806	-0.4	0.0001
		SYW	SYNW0249	greenyellow	-0.957	-0.6	0.029
K00030	isocitrate dehydrogenase (NAD+)	PMM	PMM1596	magenta	0.714	0.4	0.008
		PMT	PMT1935	turquoise	-0.611	-0.2	0.006
		SYG	sync_0214	pink	0.757	-0.5	0.002
		SYW	SYNW0166	yellow	-0.964	-0.5	0.001
K03588	cell division protein FtsW	PMM	PMM1458	green	0.661	-0.6	0.023
		PMT	PMT1475	turquoise	-0.611	-0.2	0.027
		SYG	sync_2326	turquoise	-0.806	-0.3	0.012
		SYW	SYNW0475	yellow	-0.964	-0.2	0.011
K05371	phycocyanobilin:ferredoxin oxidoreductase	PMM	PMM0747	cyan	0.743	-0.3	0.0003
		PMT	PMT0590	pink	0.695	0.3	0.026
		SYG	sync_1656	turquoise	-0.806	-0.4	0.0011
		SYW	SYNW1084	brown	0.869	0.2	0.048618
K01299	carboxypeptidase Taq (M32) metallopeptidase	PMM	PMM0493	red	-0.813	-0.8	0.015
		PMT	PMT1279	greenyellow	-0.955	-0.3	0.0005
		SYG	sync_2040	purple	-0.808	-0.4	0.0008
		SYW	SYNW1098	greenyellow	-0.957	0.3	6.78E-05
---	LysM domain	PMM	PMM0330	turquoise	-0.755	0.7	0.003
		PMT	PMT0190	turquoise	-0.611	-0.2	0.004
		SYG	sync_0565	brown	0.977	-0.7	2.19E-05
		SYW	SYNW1957	yellow	-0.964	-0.3	0.0007
---	ABC transporter, ATP binding component	PMM	PMM0290	red	-0.813	0.4	0.002
		PMT	PMT1635	turquoise	-0.611	-0.3	0.005
		SYG	sync_2588	darkturquoise	-0.746	-0.2	0.0207
		SYW	SYNW0320	black	0.791	0.7	0.015
K02639	ferredoxin	PMM	PMM0898	red	-0.813	-0.1	0.035
		PMT	PMT1429	purple	0.613	0.6	0.011
		SYG	sync_1953	turquoise	-0.806	-0.6	0.006
		SYW	SYNW1277	midnightblue	-0.722	0.4	0.012

Several other stress-regulated genes detected in all four selected cyanobacteria species also showed different direction of regulation (*i.e.* up- or down-regulation) in all species. For instance, ABC transporter ATP binding component [50] was up-regulated in PMM and SYW but down-regulated in PMT and SYG; carboxypeptidase Taq metallopeptidase was up-regulated in SYW but down-regulated in the other 3 species. Isocitrate dehydrogenase (NAD^+^) (*idh3*), involved in several metabolism pathways like citrate cycle and secondary metabolite biosynthesis, was up-regulated in PMM but down-regulated in PMT, SYG and SYW. Similar to *idh3*, LysM domain gene encoding a LysM-containing protein involved in bacterial cell wall degradation [51], was also up-regulated in PMM but down-regulated in PMT, SYG and SYW. Interestingly, gene encoding PurH catalyzing the last two steps of *de novo* purine biosynthesis in bacteria and eukaryotes [52], was up-regulated in iron depletion but down-regulated in copper toxicity treatments.

Phycocyanobilin:ferredoxin oxidoreductase, PcyA, catalyzes biosynthesis of the phycobili-protein and phytochrome chromophore precursor phycocyanobilin [53]. Phycocyanobilin can serve as a light-harvesting pigment in the photosynthetic light-harvesting structures of cyanobacteria called phycobilisomes [54]. Its encoding gene was found up-regulated in PMT and SYW but down-regulated in PMM and SYG.

Ferredoxin coding gene was up-regulated in PMT and SYW but down-regulated in PMM and SYG. Level of cellular ferredoxin and flavodoxin was previously proposed as an indicator of iron stress in cyanobacteria [55]; however, a recent analysis about iron availability and production of ferredoxin in Antarctic sea ice diatoms showed no strong correlation between their levels and stress conditions [56]. Our results here supported the argument that ferredoxin level alone may not be a strong evidence of iron limited growth.

Another gene responsive in both iron and copper perturbations was *pts* encoding an enzyme involved in the early biosynthetic pathway of pteridines in cyanobacteria [57]. The gene was found up-regulated in species of PMT, SYW and SYG. The role of cyanopterin in UV/blue light single transduction of cyanobacterium *Synechocystis* sp. PCC 6803 phototaxis was verified recently [58]. Cyanobacteria are well known for producing high amounts of pteridine glycosides, and pterin compounds have been suggested as possible photoreceptor pigments in some stress responses that are induced by UV and blue light, probably due to their chemical and photophysical properties [59].

With the aid of WCGNA network analysis, it is also possible to obtain novel insights into molecular responses to environmental changes which will otherwise not be achieved by analyzing data from a single species. For example, a total of 27 shared metal-responsive KEGG pathways between two metal stresses were determined by the WGCNA analysis (Additional file 11: Table S7), which included 20 pathways of “amino acid, nitrogen, sulfur metabolism”, 2 pathways of “photosynthesis and porphyrin and chlorophyll metabolism”, 1 pathway each for “ABC transporters”, “oxidative phosphorylation”, “DNA mismatch repair”, and “biosynthesis of secondary metabolites”, respectively. Based on the fact that they responded to both iron depletion and high copper toxicity in multiple cyanobacteria species, we proposed that these pathways may represent the core metal-responsive pathways. Moreover, among all 40 signature genes of the PMT-greenyellow module, many were involved in amino acid metabolism, secondary metabolites, ABC transporters, and homologous recombination. PMM, originally isolated from surface water of the Mediterranean ocean, is high-light adapted with higher copper tolerance [25]. Probably due to these physiological differences, some highly correlated modules detected in PMT were not seen in the marine coastal species PMM, although a large number of active KEGG pathways detected were commonly shared by two species under the iron depletion condition (Table 2). It has been speculated that the better adaptation of PMT to lower iron concentration may be due to more efficient iron transport systems, better protection of iron stress and lower requirement for iron during growth [25].

### Divergent hub genes for different cyanobacteria species under metal stress

Hub genes which have high connectivity within the networks are considered to play important roles in different phenotypes, such as yeast viability [60] and drought stress [61]. Recent work on cyanobacteria showed that there existed big divergence of gene expression patterns between cyanobacteria species from different ecological environments [25,31].

The genes might be considered as hub genes to a specific stress with the most connected genes in the responsive modules developed from the WGCNA analysis [60,61]. One of the major goals of this cross-species analysis was to discover the hub genes in each cyanobacteria species under specific environmental stimuli. The WGCNA analysis showed that some genes were connected with more than 8 other genes. Based on the annotated genes in the WGCNA modules, we obtained modest lists of orthologous hub genes for PMT responsive to iron depletion (number of the genes: 16) and SYW to copper toxicity (16), and shorter lists for PMM (10) and SYG (6). Surprisingly, no overlap of the hub genes was found between species, suggesting they may be unique to each species. For example, in PMT, the hub genes mainly encode metabolic enzymes, like GMP synthase (glutamine-hydrolysing), ketol-acid reductoisomerase; while in PMM, the genes were involved in photosynthesis system (*psaI* and *rbcL*) and two-component system (*phoB*). Nearly half of the hub genes in SYW encode ribosomal proteins (7) and oxidative phosphoraylation proteins (*atpf1a* and *atpf1d*), compared to SYG hub genes with functions of metabolic enzymes and photosysm II PsbK protein and phycoerythrin-associated linker proteins (Additional file 12: Table S8). In terms of the functionally unknown genes in the modules (Additional file 13: Table S9), the analysis showed that they were also included in some gene clusters. For instance, PMT101-102-107 (black module), PMM1395-1396 (green module), sync_2434-2436 (purple module) were grouped together, while SYWN0193-0194 and SYWN0319-0320 were not in the same module. The function of these stress-responsive gene clusters with unknown functions may worth further investigation [60].

**Figure 1:**
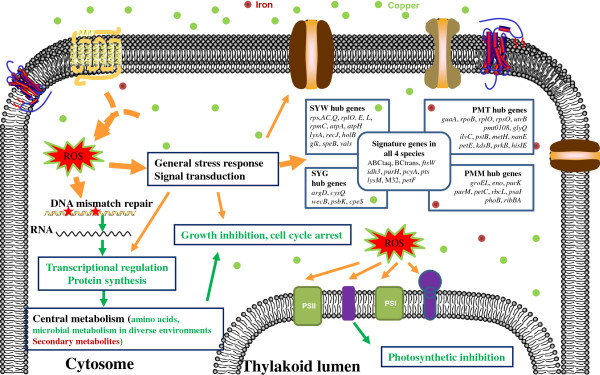
**Schematic representation of responsive genes regulated by metals in cyanobacteria.** See text for details, based on WGCNA network analysis in this study and previous reports.

Hub genes obtained through the WGCNA analysis also showed distinct genes involved in iron stress in different *Prochlorococcus* species, which provided important clues for the differential fitness of these two species at low iron concentrations: *i*) the existence of a unique gene, lipopolysaccharide transport system ATP-binding protein (ABC-2.LPSE.A) and the activation of a urea transport system permease protein (*urtB*) may cause a more efficient iron transport features in PMT; *ii*) down-regulated ribosome genes (*rp-S15*, *rp-L3*) in PMT could save energy for cell survival under low iron concentration; and *iii*) differentially expressed hypothetical genes with unknown functions in some of the highly correlated modules, especially those in PMT, may play important roles in the differential fitness [62]. Based on the combined analysis of both modules and hub genes, our study indicated that different *Prochlorococcus* species employed some specific metabolic pathways, together with general stress responses, to survive in different environments. Based our WGCNA analysis results and a previous review [63], the representation of responsive networks regulated by metals in cyanobacteria was schemed in Figure 1.

## Conclusions

In the study, we have provided evidences that cross-species WGCNA analysis could be a powerful tool to investigate the molecular mechanisms underlying the response of microbes to their environments. Based on the shared genes identified between all 4 cyanobacteria species evaluated in this study, we have found some similar strategies adopted by the cyanoabcteria to deal with different stresses. Further investigation of these shared genes showing differential expression in various species may shed light on their adaption in unique environment. In addition, our cross-species analysis has found some obvious differences between two metal treatments. The extraction of hub genes highly connected with other genes in cyanobacteria genomes may be valuable in determining essential genes for cyanobacteria [64]. Finally, we also noticed many hypothetical genes with function unknown were grouped with important genes in some stress-correlated modules. Although they were not discussed in details in this paper due to the fact of no much functional information is available for them, we believe that further deciphering their functions will provide new insights to the stress responses in cyanobacteria.

## Methods

### Dataset acquisition

Microarray datasets of four cyanobacteria species, *Prochlorococcus* MED4 (PMM), *Prochlorococcus* MIT9313 (PMT), *Synechococcus* 9311 (SYG) and *Synechococcus* WH 8102 (SYW) [25,31], were downloaded from the Gene Expression Omnibus (GEO) [65] (Table 1). The experiments were designed for with iron (1 μM) as control and without iron as treatment for *Prochlorococcus* at different time (0, 12, 24 and 48 h for PMM and 0, 26, 28 and 53 h for PMT based on their growth stages) and 2 h of copper treatments with concentrations of 1 μM (SYW) and 0.1 μM (SYG) [25,31]. The experimental details can be found in the original publication [25,31]. To compile an extensive set of comparable data, we collected all relevant datasets from the two experiments and then removed the outliers or samples forming an out-group to ensure the results reflecting the real biological response to different treatments. Specifically, we first performed a hierarchical clustering analysis (ward method) using (1-r) as distance for all samples, where r is the pair-wise (between any two pairs of samples) correlation coefficient for the expression of all genes (probesets). Then we compared the tree structure with the status of treatment condition. For samples contradicting with the status of treatment, we excluded them from the downstream network analysis. For example, we found that in both *Prochlorococcus* datasets, all samples at 0 hour clustered together no matter they were depleted with iron or had iron, suggesting that their expression was irrelevant to iron status. Including them will compromise the power of the analysis. Therefore, we excluded all samples at 0 hour from our analysis. The updated genome annotation for all four species was downloaded from NCBI and the Comprehensive Microbial Resource (CMR) of TIGR (http://www.tigr.org/CMR) [32-34]. KEGG Pathway and COGs (Cluster of Orthologous Groups of proteins) information was obtained from the KEGG Pathway Databases (http://www.genome.jp/kegg/pathway.html) and the COG Database of NCBI (http://www.ncbi.nlm.nih.gov/books/NBK21090/).

### Network construction

**Figure 2:**
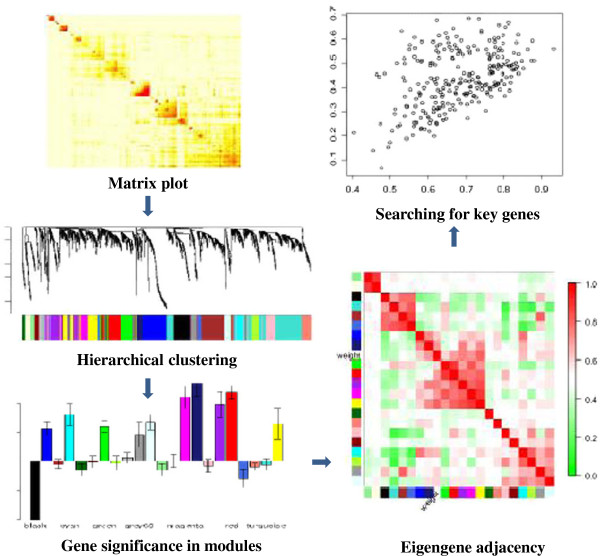
**Scheme of the WGCNA work flow used in this study.** Details of the WGCNA work flow referred in the text and reference (13).

For each cyanobacteria species, we created a transcriptional network from the microarray data, first by calculating weighted *Pearson* correlation matrices corresponding to gene expression, and then by following the standard procedure of WGCNA to create the networks [13]. Figure 2 schemes the methodology used in this study. Briefly, weighted correlation matrices were transformed into matrices of connection strengths using a power function [13]. These connection strengths were then used to calculate topological overlap (TO), a robust and biologically meaningful measurement that encapsulates the similarity of two genes’ co-expression relationships with all other genes in the network [13]. Hierarchical clustering based on TO was used to group genes with highly similar co-expression relationships into modules. From the expression data we followed the protocols of WGCNA [60,66] to create within-species consensus networks. Gene dendrograms were obtained by average linkage hierarchical clustering (Additional file 14: Figure S5, A-D), while the color row underneath the dendgram showed the module assignment determined by the Dynamic Tree Cut (WGCNA). The network for each module was generated with the minimum spanning tree with dissimilarity matrix from WGCNA. To focus on biological interpretation, in this study, only genes with functional annotation were included. The modules with *r* > 0.6, and *p*-value less than 0.05 were extracted for further investigation. KEGG pathway analysis was applied to detect the common pathways involved among different modules. Hub genes were screened by the links (≥ 8) in the modules strongly associated with phenotype (iron depletion or copper toxicity, based on correlation coefficient *r* > 0.6).

To establish cross-species signature genes in this analysis, and to define conservation in terms of stress response across cyanobacteria species, phenotype-correlated modules and the associated genes in each species were extracted. To assess co-expression perseveration between the species on a module-by-module basis, we first calculated a value of the module membership (MM) - a measure of how well each gene correlates with the first principal component of gene expression within a module, termed as the module eigengene. We then imposed a threshold based on MM values (*r*, *p*) to make the final module assignments.

## Abbreviations

ABC: ATP-binding cassette;FtsW: Cell division protein W;FtsQ: Cell division protein Q;HK: Histidine kinase;Idh3: Isocitrate dehydrogenase (nad^+^);kegg: Kyoto encyclopedia of genes and genomes;pcya: Phycocyanobilin:ferredoxin oxidoreductase;pts: 6-pyruvoyl tetrahydrobiopterin synthase;pmm: *Prochlorococcus* med4;pmt: *Prochlorococcus* mit9313;purh: Phosphoribosylaminoimidazolecarboxamide formyltransferase/imp cyclohydrolase;rr: Response regulator;syg: *Synechococcus* 9311;syw: *Synechococcus* wh 8102;tca: Citrate cycle;urtb: Urea transport system permease protein;WGCNA: Weighted Gene Co-expression Network Analysis

## Competing interests

The authors declare that they have no competing interests.

## Authors’ contributions

JW, GW, LC and WZ were responsible for the study design and coordination, contributed to the bioinformatics analysis and wrote the manuscript. JW and GW performed WGCNA and were responsible for the biological analysis. LC contributed to the data acquisition. All authors read and approved the final manuscript.

## Supplementary Material

Additional file 1: Figure S1 Clustering of the transcriptomic datasets under iron and copper treatments in different species. The grouped datasets with solid red color in “HighCopper” and “Iron” suggested high confident grouping of treated groups from controls. Click here for file

Additional file 2: Figure S2 Association between phenotypes and identified transcriptional modules in different species. Each of the identified transcriptional modules was indicated by different colors, and their association with the phenotypes was indicated by *p*-values. Click here for file

Additional file 3: Figure S3 Overlap of the detected responsive modules within and between cyanobacteria species. The lines connected different cyanobacteria species means there are some genes shared between specific modules. Click here for file

Additional file 4: Table S1 genes and pathways in SYW-yellow module. Click here for file

Additional file 5: Table S2 genes and pathways in SYW-Green yellow module. Click here for file

Additional file 6: Table S3 genes and pathways in SYG-dark green module. Click here for file

Additional file 7: Table S4 genes and pathways in SYG-brown module. Click here for file

Additional file 8: Figure S4 Shared responsive genes in cyanobacteria species to iron depletion and copper toxicity treatments, respectively, and shared responsive genes between treatments in all cyanobacteria species. Nine shared genes among all 4 cyanobacteria species were listed in Table 3. Click here for file

Additional file 9: Table S5 shared genes in iron treatments. The color of each gene indicates differential change: red is up-regulated and green is down-regulated. Click here for file

Additional file 10: Table S6 shared signature genes in copper treatments. The color of each gene indicates the differential change, red is up-regulated and green is down-regulated. Click here for file

Additional file 11: Table S7 Pathways shared in all 4 species. Click here for file

Additional file 12: Table S8 Hub genes with known functions in different cyanobacteria species. The same color from KO_ID and the gene names indicate the hub genes in each species. Click here for file

Additional file 13: Table S9 Hub genes with unknown functions in different cyanobacteria species. Click here for file

Additional file 14: Figure S5 Hierarchical clustering tree using the topological overlap dissimilarity. Tree branches have been colored by module membership. A, B) PMT, PMM to iron, respectively; C, D) SYW, SYG to copper, respectively. Please refer the text for details of the analysis. Click here for file
