# Supplementary figures and images for: Cross-species transcriptional network analysis reveals conservation and variation in response to metal stress in cyanobacteria
Source: BMC Genomics. 2013 Feb 19;14:112. doi: 10.1186/1471-2164-14-112 (PMC3598940; doi:10.1186/1471-2164-14-112)

## Slide 1
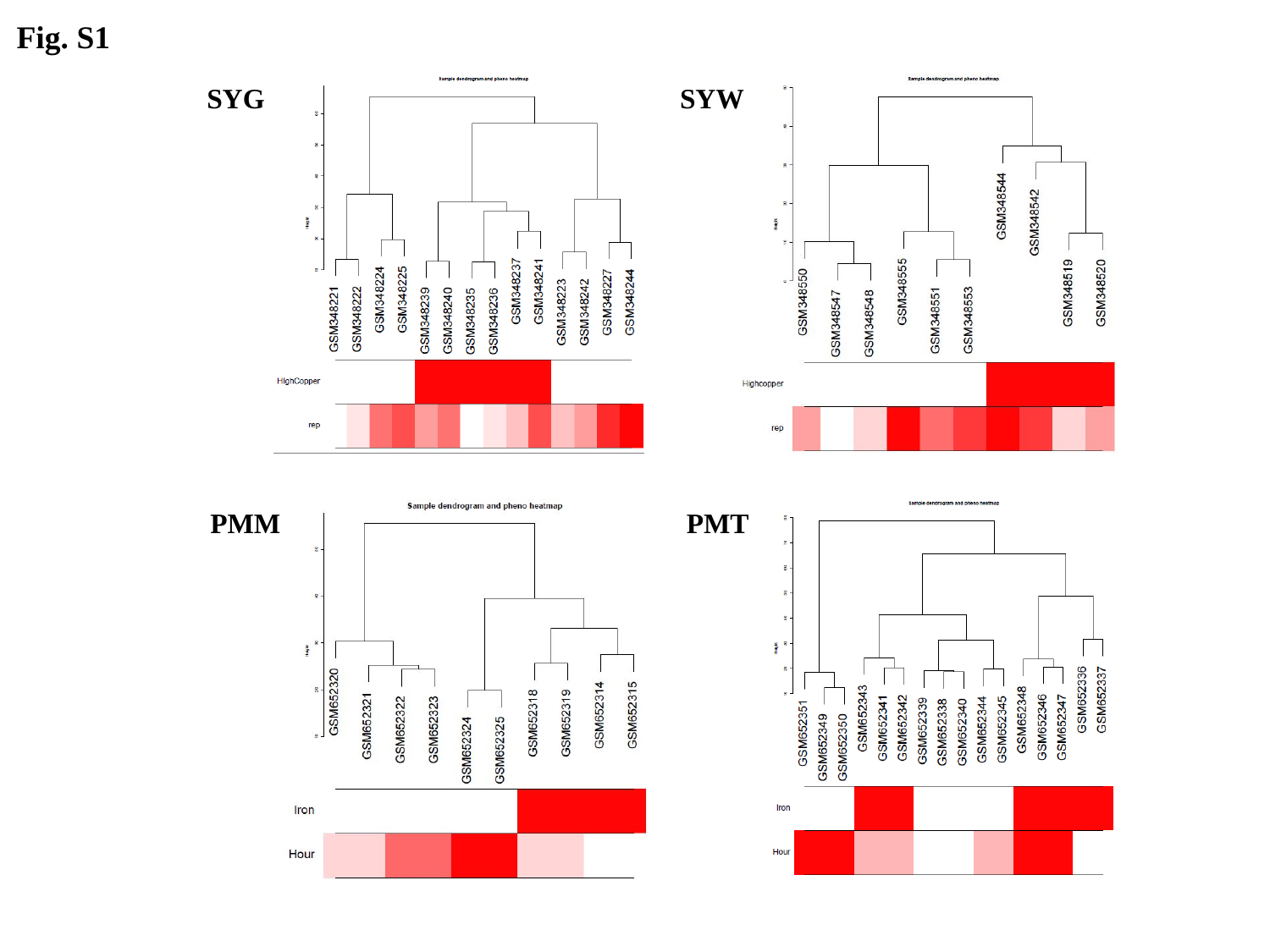

Fig. S1
SYG
SYW
PMM
PMT

Supplement: Additional file 1: Figure S1 — Clustering of the transcriptomic datasets under iron and copper treatments in different species. The grouped datasets with solid red color in “HighCopper” and “Iron” suggested high confident grouping of treated groups from controls. [file 1471-2164-14-112-S1.ppt]

## Slide 1
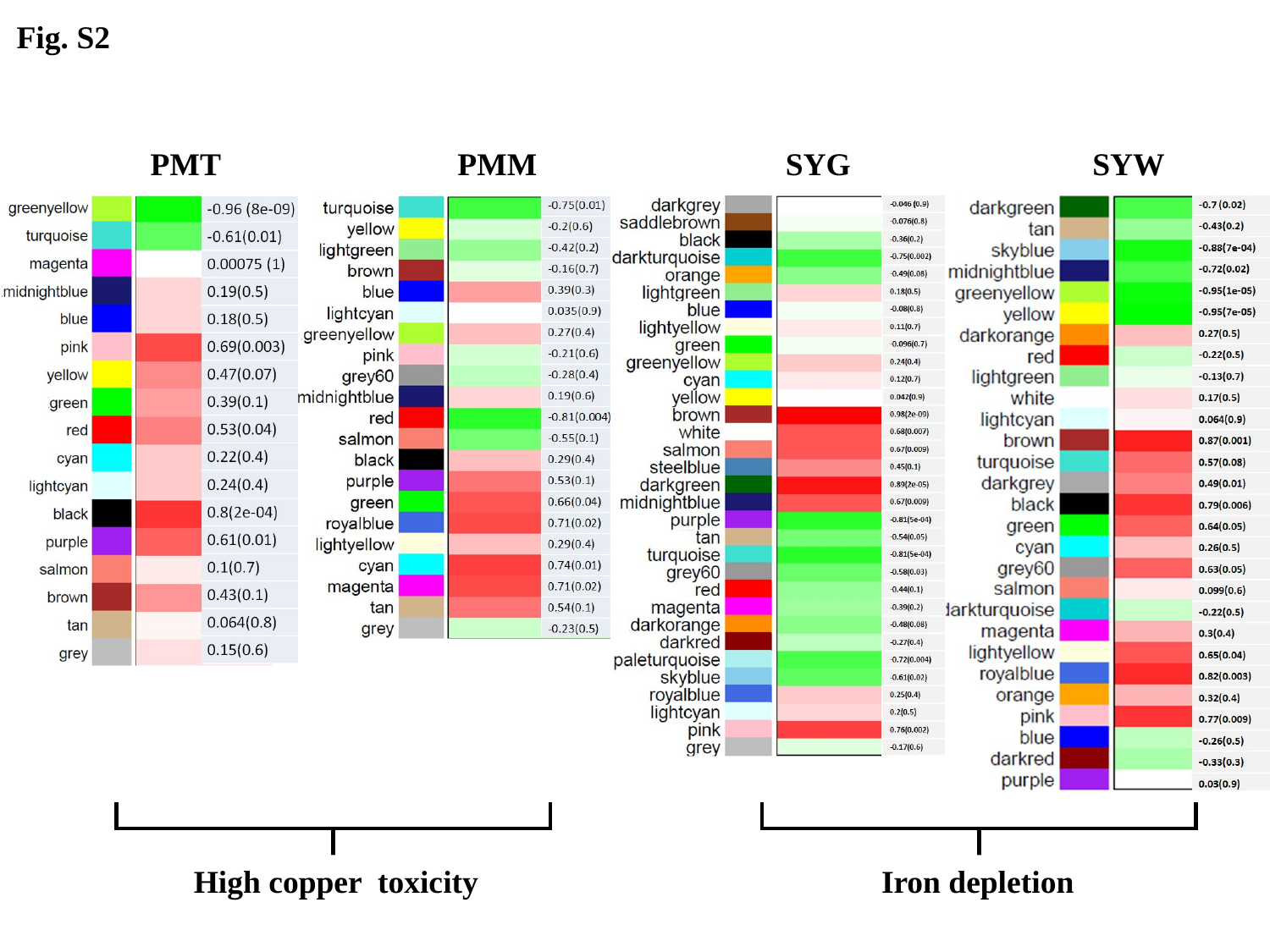

Fig. S2
PMT
PMM
SYG
SYW
High copper toxicity
Iron depletion

Supplement: Additional file 2: Figure S2 — Association between phenotypes and identified transcriptional modules in different species. Each of the identified transcriptional modules was indicated by different colors, and their association with the phenotypes was indicated by p-values. [file 1471-2164-14-112-S2.ppt]

## Slide 1
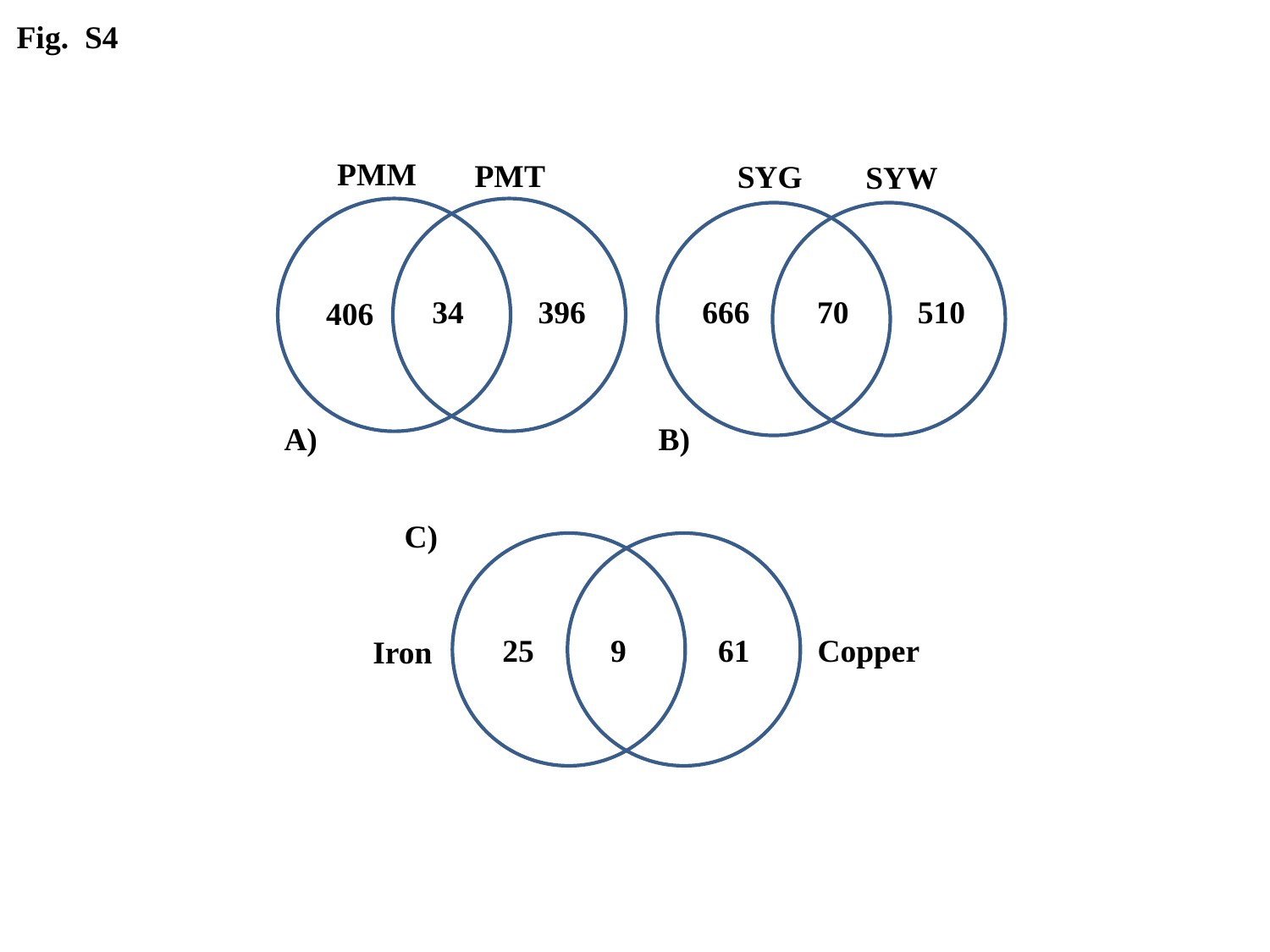

Fig. S4
PMM
PMT
34
396
406
SYG
SYW
666
70
510
A)
B)
C)
25
9
61
Copper
Iron

Supplement: Additional file 8: Figure S4 — Shared responsive genes in cyanobacteria species to iron depletion and copper toxicity treatments, respectively, and shared responsive genes between treatments in all cyanobacteria species. Nine shared genes among all 4 cyanobacteria species were listed in Table 3. [file 1471-2164-14-112-S8.ppt]

## Slide 1
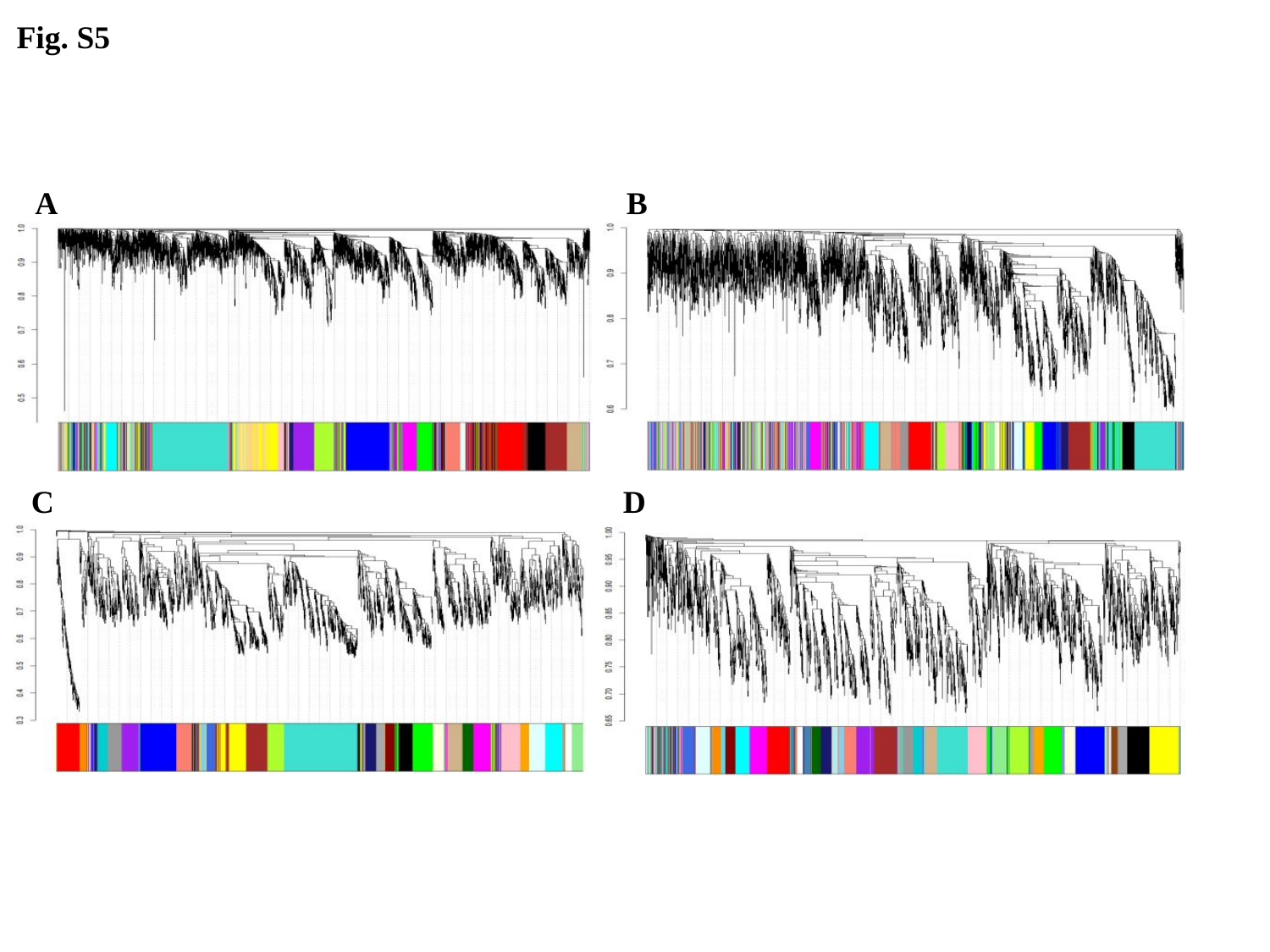

Fig. S5
A
B
C
D

Supplement: Additional file 14: Figure S5 — Hierarchical clustering tree using the topological overlap dissimilarity. Tree branches have been colored by module membership. A, B) PMT, PMM to iron, respectively; C, D) SYW, SYG to copper, respectively. Please refer the text for details of the analysis. [file 1471-2164-14-112-S14.ppt]
