# Supplementary material for: Cross-species transcriptional network analysis reveals conservation and variation in response to metal stress in cyanobacteria
Source: BMC Genomics. 2013 Feb 19;14:112. doi: 10.1186/1471-2164-14-112 (PMC3598940; doi:10.1186/1471-2164-14-112)
Supplement: Additional file 3: Figure S3 — Overlap of the detected responsive modules within and between cyanobacteria species. The lines connected different cyanobacteria species means there are some genes shared between specific modules. [file 1471-2164-14-112-S3.ppt]

## Slide 1
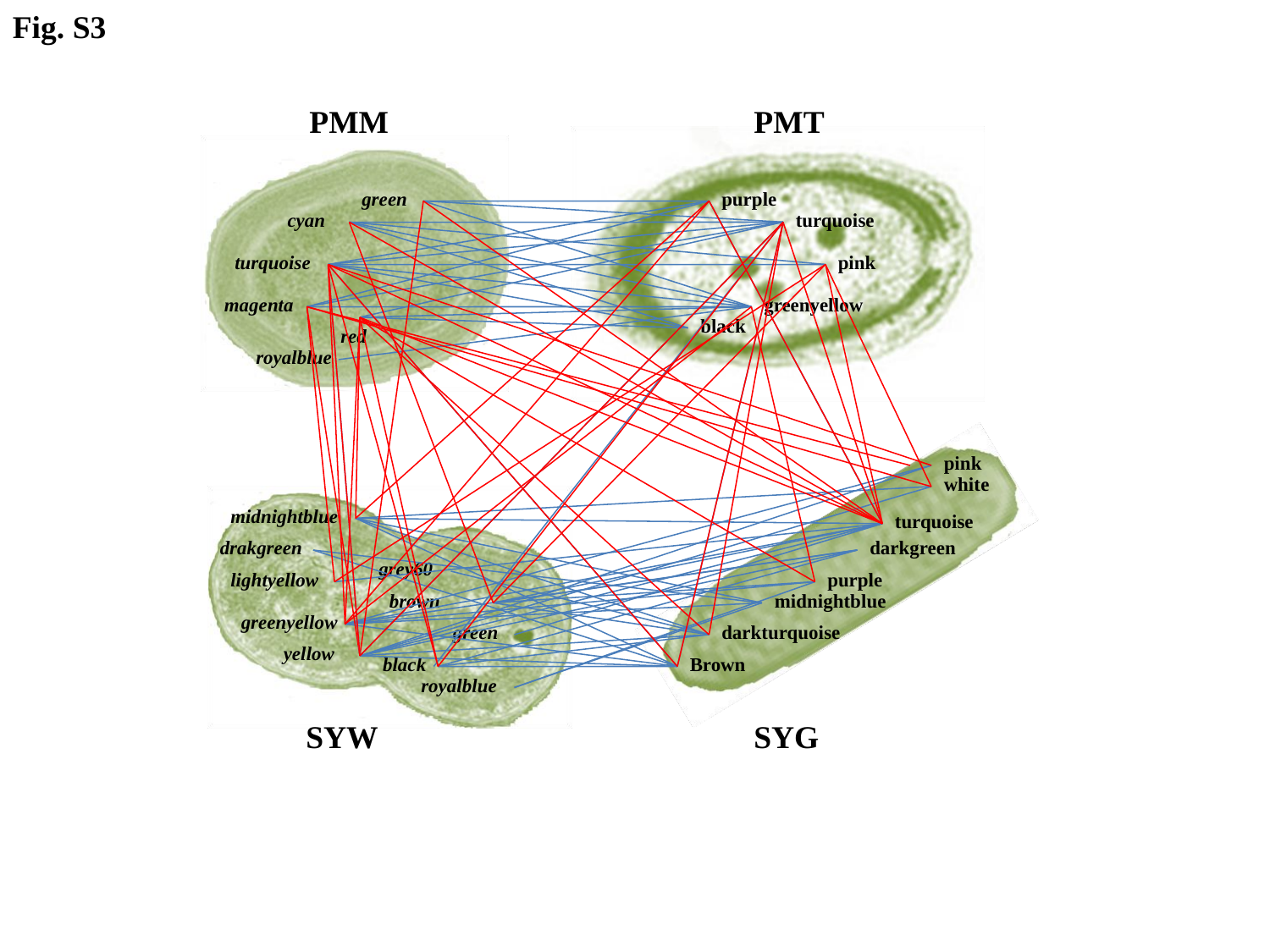

Fig. S3
PMM
PMT
green
purple
cyan
turquoise
turquoise
pink
magenta
greenyellow
black
red
royalblue
pink
white
midnightblue
turquoise
drakgreen
darkgreen
grey60
lightyellow
purple
brown
midnightblue
greenyellow
green
darkturquoise
yellow
black
Brown
royalblue
SYW
SYG
